# ‘*Why Didn't I Get That Choice?’*: A Qualitative Exploration of How Cervical Screening Choices Are Understood and Experienced by Screen‐Eligible People in Australia, Two Years After Self‐Collection Became an Option for All

**DOI:** 10.1111/hex.70397

**Published:** 2025-08-19

**Authors:** Ana Machado Colling, Tessa Saunders, Nicola Creagh, Maleeha Ashfaq, Julia Brotherton, Claire Nightingale

**Affiliations:** ^1^ Evaluation and Implementation Science Unit, Centre for Health Policy, Melbourne School of Population and Global Health The University of Melbourne Melbourne Australia

**Keywords:** cervical screening, choice, decision, implementation, information, qualitative, self‐collection

## Abstract

**Background:**

Australia's human papillomavirus (HPV)‐based National Cervical Screening Program guidelines state that anyone eligible for screening should be offered a choice of using self‐collection or clinician collection for initial screening.

**Aim:**

To explore the awareness and experiences of women and people with a cervix who have been screened since this choice became available in July 2022.

**Methods:**

Semi‐structured online or telephone interviews were conducted between February and May 2024 with 43 Victorian women and people with a cervix, aged 25–74 years. All participants self‐reported completing a cervical screen within 12 months of our study. Interview data were recorded, transcribed verbatim and thematically analysed before being mapped to The Ottawa Decision Support Framework. Self‐reported screening history was confirmed with de‐identified data from the National Cancer Screening Register (NCSR) Victorian Raw Data Extract (November 2024).

**Results:**

Fewer than half (19, 44%) of those interviewed were given a choice at their most recent screen, with variation in how options were presented by healthcare providers. Participants felt they lacked awareness and knowledge to feel confident in their options.

Most participants viewed having a choice as important and, even if they did not prefer self‐collection for themselves, noted benefits for others. Some felt disappointed or angry about not having a choice, while others were happy to defer to their doctor.

Relationships with, and the views of, healthcare providers strongly influenced decision‐making. Participants reflected on potential advantages of self‐collection if it could reduce the cost of appointments and be accessed in more flexible ways.

Among the 38 participants who consented to screening history verification, the self‐reported data showed reasonable accuracy (67%) against the NCSR.

**Conclusion:**

Despite a clear policy directive for practitioners to offer a choice to all eligible individuals, many recent screeners were not offered the choice or lacked the knowledge, confidence and decision supports needed to make an informed choice. The choice of screening method appears strongly influenced by if, and how, options are presented by healthcare providers. A range of strategies are needed to ensure screeners feel empowered, supported and informed to make and carry out a real choice.

**Patient or Public Contribution:**

Members of the public were involved in interviews. Findings were summarised and disseminated via a short report. A consumer advisory panel provided feedback on the content, readability and length of all patient‐facing resources.

## 1 Introduction

Australia's National Cervical Screening Program (NCSP) recommends 5‐yearly human papillomavirus (HPV)‐based screening for women and people with a cervix aged 25–74. Under the NCSP guidelines, anyone eligible for screening should be offered a choice between self‐collection (whereby a person can use a small swab inserted into the low‐mid vagina to collect the specimen) or clinician collection for screening [1]. The guidelines state that clear information should be provided to support informed decision‐making about these options.

Australia's current model of care requires that self‐collection is ordered and overseen by a healthcare provider, with general practitioners (GPs), nurse practitioners (NPs) and other specialist providers authorised to sign the pathology request for tests reimbursable under the current Medicare Benefits Schedule (MBS) regulations [1].

In 2022, self‐collection became an option for everyone eligible for primary screening and for HPV detection in follow‐up [2]. For 4 years prior, its use had been restricted to under‐screened people. Because self‐collection must be accessed through a healthcare provider in Australia [1], this choice is dependent on discussion between the individual and the healthcare provider. The NCSP guidelines emphasise taking a ‘person‐centred’ approach to presenting this choice, where person‐centred is understood as ‘seeking out and understanding what is important for each screen‐eligible person, fostering trust, establishing mutual respect, and enabling shared decision‐making’ [1]. Shared decision‐making is key to enabling informed choices about screening options and is defined within the literature as a ‘patient centred approach to care in situations where there is more than one medically reasonable option … and [can] prove beneficial in situations where more than one treatment or screening decision is valid’ [3].

**Figure 1:**
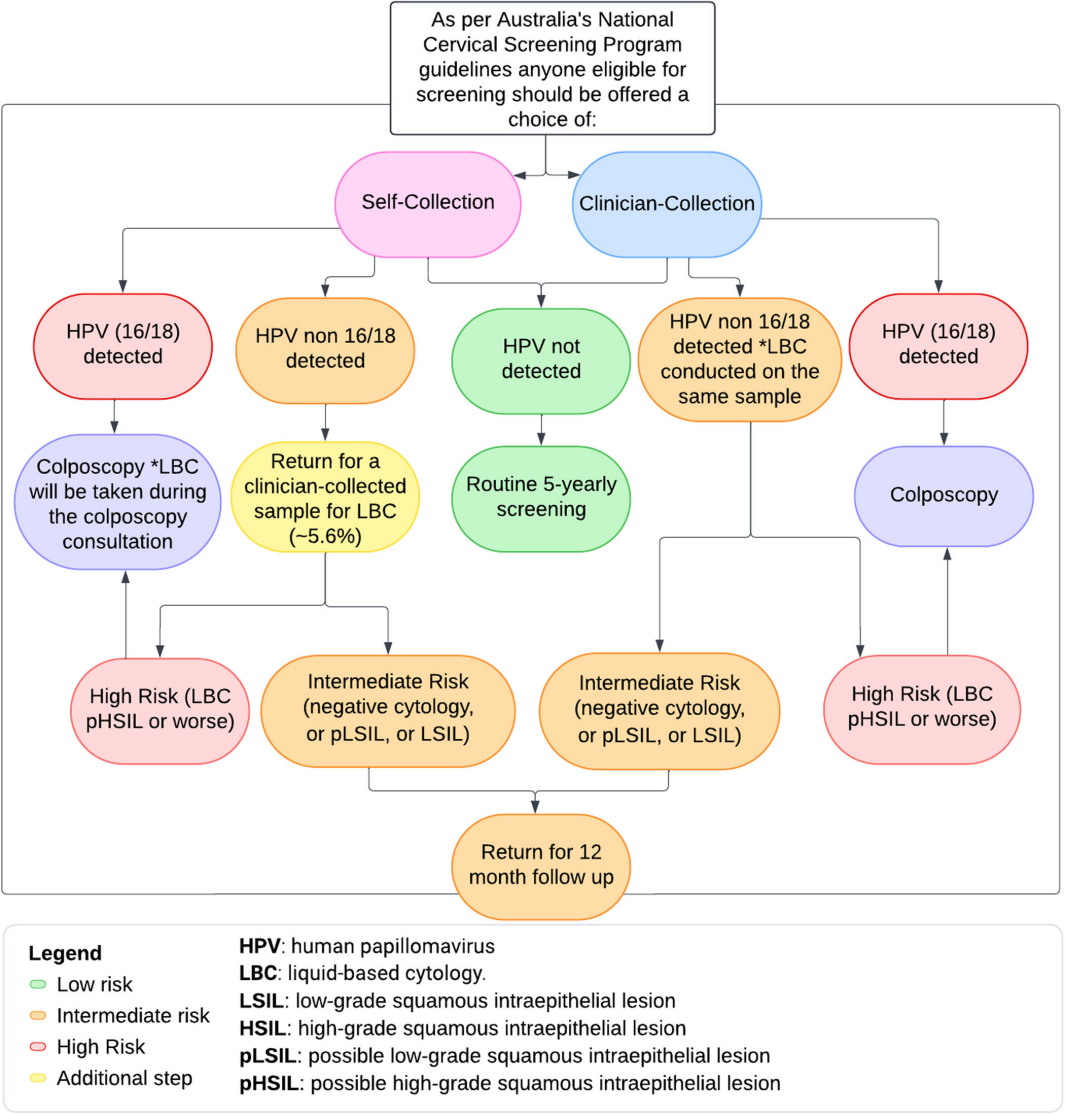
Cervical Screening Results Management Pathway, adapted from Cancer Council Australia^1^ [5].

There are some key considerations in making the choice between clinician collection and self‐collection and these include (a) client preference between the options, noting that a clinician‐collected test requires the use of a speculum (b) if using self‐collection, there is the potential to be recalled for an additional visit to have a cervical sample collected if HPV non‐16/18 is detected on the initial sample (Figure 1), which occurs in approximately 5.6% of all screening cases but the range of positivity changes considerably by age, ranging from 17.3% in individuals aged 25–29 to 2.2% in those aged 70–74 [4] (if a cervical sample is initially collected, this is automatically reflexed to cytology in the laboratory) (c) the slightly higher risk of an unsatisfactory specimen with self‐collection, which is 1%–2% versus < 0.2% for clinician‐collection [5]. An individual can also choose to have an assisted self‐collection (from the low‐mid vagina) by a health professional if they prefer [1].

Through the transition to HPV screening and initial introduction of restricted‐access self‐collection, awareness and adoption of self‐collection remained limited. Many healthcare providers held concerns about the accuracy of self‐collection, perceptions of missed opportunities to visualise the cervix, and confusion over screening guidelines and how to access laboratory services to process the specimen [6, 7, 8, 9, 10]. Further, while most studies find self‐collection to be highly acceptable and preferable among screen‐eligible people [11, 12], concerns about the accuracy and how to perform self‐collection were commonly reported [11, 13].

While the proportion of self‐collected primary screens has been steadily increasing since 2022, significant variation across states and territories has been observed [14]. As of June 2024, approximately 44% of cervical screens in Victoria were self‐collected, slightly higher than the national average of 37% [15]. In contrast, New Zealand, which also follows a practitioner‐centred delivery model but introduced universal self‐collection from the outset of the change to HPV in 2023, has witnessed a higher uptake, exceeding 80% in the first year of its availability [16]. The differences in the use of self‐collection in Australia may reflect a variation in its delivery by healthcare providers, with a recent study highlighting that not all providers offer an equal choice between self‐collection and clinician collection [17]. However, to date, there have not been any studies exploring how screening participants have experienced the implementation of a choice of screening options. To address this gap, we aimed to explore how cervical screening choices were presented to, and understood by, recent screeners in Victoria, Australia, 2 years after self‐collection became available as an option for all.

## 2 Methods

We employed a qualitative research design utilising semi‐structured interviews to explore how cervical screening choices were experienced by recently screened people in Australia. The Ottawa Decision Support Framework (ODSF) [18] informed the methodological approach. The ODSF identifies five decisional needs that, if unmet, can impact the quality of a decision: decisional conflict (uncertainty), knowledge of options, expectations and values, beliefs about others' opinions, and access to support and resources to make and implement decisions.

### 2.1 Recruitment

We purposively recruited women and people with a cervix aged 25–74 years in Victoria, Australia, who spoke English and self‐reported a cervical screen within the 12 months before the interview. This time frame aligned with the July 2022 policy change of universal access to the choice of self‐collection and supported recall of information. Tailored recruitment materials, including flyers, social media tiles and newsletter blurbs with a QR code linking to an expression of interest (EOI) form, were distributed to potential participants through community‐based organisations, including Cancer Council Victoria, Sexual Health Victoria, the Multicultural Centre for Women's Health, public libraries and local council groups. After receiving an EOI, we confirmed the participant's interest and availability over email. During the recruitment process, we monitored participant demographics and test collection method using EOI data and undertook targeted recruitment to ensure a broader range of ages, country of birth, test collection methods and geographical representation. Data were managed using REDCap electronic data capture tools hosted at the University of Melbourne [19, 20].

### 2.2 Validating Screening History

We obtained additional optional written consent from participants to confirm their self‐reported primary screening test collection method (self‐collected or clinician‐collected) and date of last screen from the National Cancer Screening Register (NCSR). The NCSR is Australia's digital platform developed to support the NCSP and provides infrastructure for the collection, storage, analysis and reporting of screening programme data [21]. Data from the NCSR Victorian Raw Data Extract (November 2024) was provided by the Australian Centre for the Prevention of Cervical Cancer (ACPCC) on behalf of the Victorian Cancer Screening Framework (VCSF) [22]. The VCSF is a funding and governance model that guides the delivery and investment of cancer screening initiatives in Victoria, Australia. Despite individual‐level consent, state‐level privacy requirements limited us to only being able to provide Medicare numbers to ACPCC for matching and not other details (such as name or address), reducing our ability to locate and confirm all participants' screening histories (categories defined in Table 1). We did not obtain any screening results.

**Table 1:** Screening history categories.

Screening history category	Definition
On‐time	On‐time participants are defined as those who have screened within 1 year of the due date. This includes early screeners, who rescreened before the due date, and all participants aged under 26.
Overdue	Overdue participants are those who have screened after 1 year from the due date. ‘Never‐screened’ participants indicate that the most recent screen is the first‐ever screen done by the participant.
In follow‐up	In follow‐up, participants are those with previous abnormalities or HPV‐positive tests and have not yet returned to routine screening intervals.

Using a secure SharePoint drive, the research team was provided with a de‐identified summary of each participant's recent screening date, test collection method and screening history, protecting privacy by using the study ID. Unmatched entries were flagged as ‘unable to match data’. Participants who requested to opt out of their data being stored on the NCSR or from the NCSP were excluded from the provided dataset.

### 2.3 Data Collection

Interviews were conducted online via Zoom or telephone based on participants' preference, by public health researchers (A.M.C., T.S., M.A. and N.C.), all of whom are cis‐gendered women experienced in qualitative research and cancer prevention. Consent was obtained via REDCap or verbally at the time of the interview. Interviews were recorded, transcribed verbatim using Otter.ai, and followed a guide informed by the ODSF. This guide explored five key themes, including awareness, perceptions and experiences of screening, choice presentation, influencing factors, and support needs. During the interview, researchers verbally provided participants with information on the screening options available under the national guidelines. The interview guide was piloted with researchers and non‐researchers. Participants could review transcripts. All received a $50 gift voucher as reimbursement.

### 2.4 Data Analysis

Before analysis, transcripts were cleaned, checked for accuracy and de‐identified. Transcripts were inductively thematically coded by authors (A.M.C., T.S. and M.A.) using NVivo 14 (QSR International Pty Ltd), to identify key themes, followed by deductive mapping of themes to the ODSF decisional needs, to understand participants' experiences and perspectives [23]. Once themes were established, a content analysis [24] was performed to quantify the instances where participants were offered a choice of screening method versus those who were not. Regular meetings with the whole research team, including senior researchers (C.N. and J.B.), were held to discuss alignment with the ODSF and to ensure that the emerging analysis reflected sufficient information power to meaningfully address the study aims [25].

### 2.5 Ethical Considerations

Ethical approval was received by the University of Melbourne Human Research Ethics Committee (HREC number 2025‐27551‐65265‐7).

Before seeking ethics approval, we gathered feedback on the content and design of all patient‐facing materials from a Consumer Advisory Panel which provides advice and guidance on our cervical screening work and includes women with disability, from culturally and linguistically diverse backgrounds, LGBTQI+ communities and with lived experience of cancer.

## 3 Results

A total of 89 EOIs were received, of which nine were suspected to be AI‐generated and five were ineligible (i.e., did not have a cervical screen within the previous 12 months or lived outside of Victoria). Of the 75 eligible EOI respondents, 4 were later excluded due to an over‐representation of the 25–34‐year age group, and 28 did not respond to two rounds of follow‐up emails.

A total of 43 women and people with a cervix participated in an interview between February and May 2024. Interviews ranged in length from 20 to 53 min (mean: 35 min). Participants were aged between 26 and 70 years (mean: 42 years). The characteristics of participants are described in Table 2.

**Table 2:** Demographic characteristics of 43 interviewed participants.

Category	Variable	*n* (%)
Age, years	25–34	15 (35)
	35–44	10 (23)
	45–54	11 (26)
	55+	7 (16)
Country of birth	Australia	31 (72)
	Overseas—English‐speaking background	3 (7)
	Overseas—non‐English‐speaking background	9 (21)
Sexual identity	Heterosexual	40 (93)
	Gay or lesbian, bisexual or used a different term	3 (7)
Education	Completed year 12 or TAFE or both	10 (23)
	Completed university degree or higher	33 (77)
Geography	Metro	32 (74)
	Rural	11 (26)
Self‐reported test collection method	Self‐collection	11 (26)
	Clinician‐collection	32 (74)

**Figure 2:**
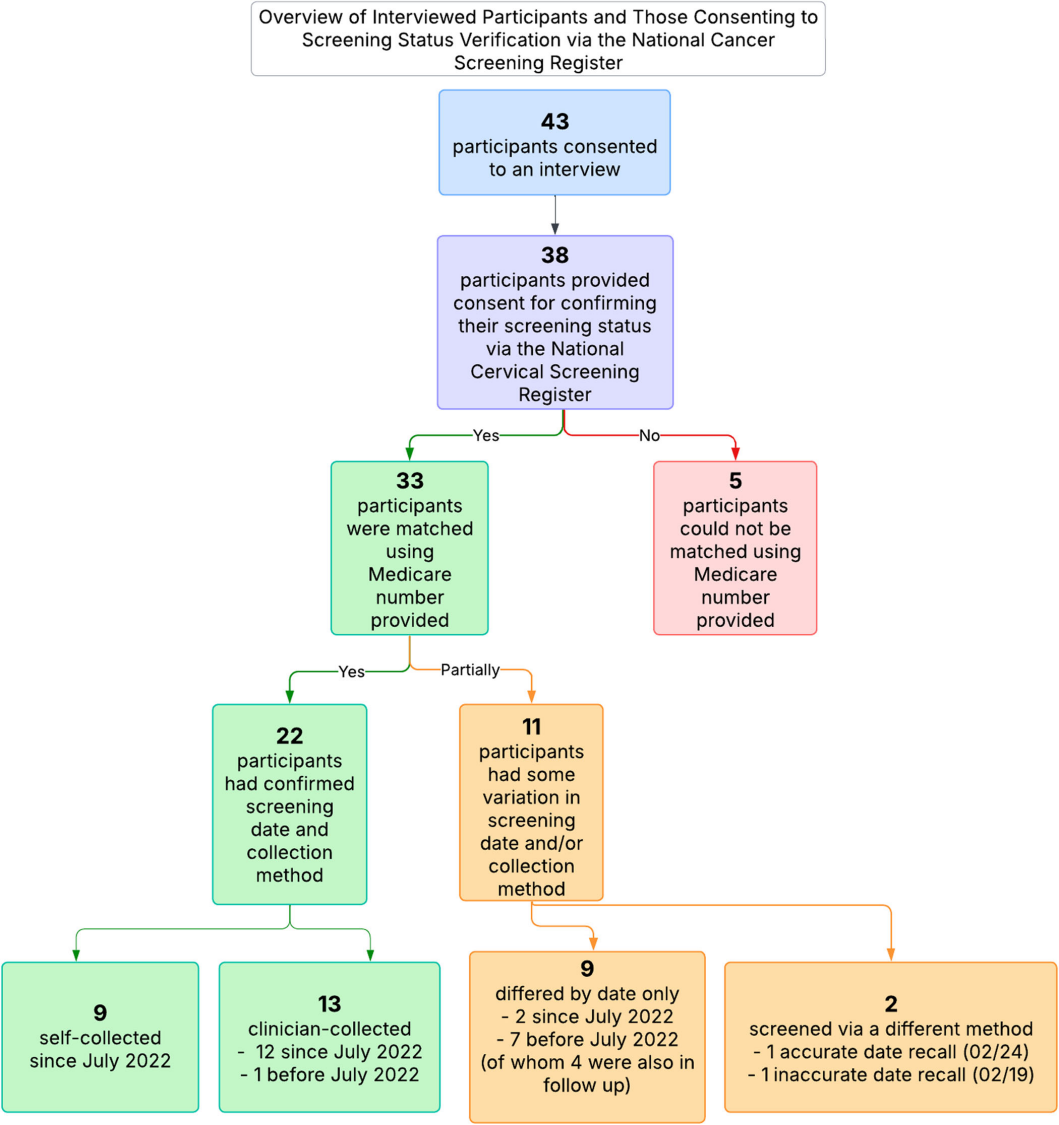
Overview of the 38 participants who provided consent for confirming their screening history via the National Cancer Screening Register.

Of the 43 participants interviewed, 38 consented to confirm their screening history against the NCSR. There was a reasonable concordance between self‐reported data and that provided via NCSR for validation (67%, *n *= 22). Variation between self‐reported and NCSR data related to discrepancies in date of last screen (9 participants, of whom 8 had screened before July 2022) and mode of collection (2 participants) (Figure 2).

**Figure 3:**
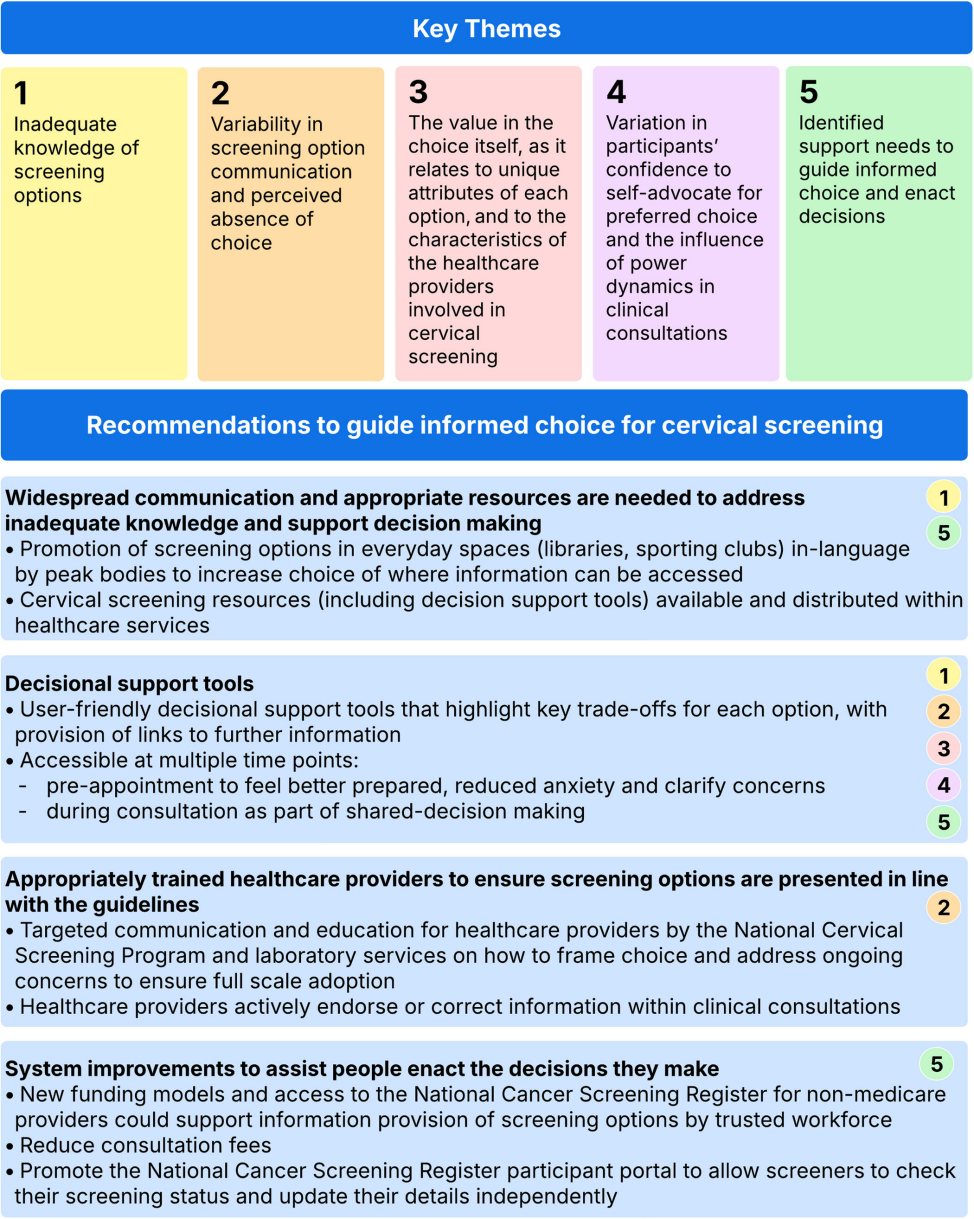
Overview of themes of the 43 interviewed screening participants with recommendations mapped to the Ottawa Decision Support Framework.

Results are presented in five main themes mapped to the ODSF (Figure 3): (1) Inadequate knowledge of cervical screening options; (2) variability in screening option communication and perceived absence of choice; (3) the value in the choice itself, as it relates to unique attributes of each option, and to the characteristics of the healthcare providers involved in cervical screening; (4) variation in participants' confidence to self‐advocate for preferred choice and the influence of power dynamics in clinical consultations and (5) identified support needs to guide informed choice and enact decisions.

### 3.1 Theme 1: Inadequate Knowledge of Cervical Screening Options

While many participants knew cervical screening options existed, most had only heard about self‐collection informally and described the information surrounding it as ‘vague’, ‘hearsay’ or ‘something in the ether’. As such, the depth of knowledge about self‐collection was low, with limited or incorrect understanding of the eligibility requirements, its accuracy and how to access self‐collection.

‘I didn't know that there was an option for me to do it myself’

ID26

Misconceptions about self‐collection were held by participants or observed in their broader community, including assumptions that it still required a speculum, had to reach the cervix, or was introduced primarily as a cost‐saving tool.

‘When I've spoken to people [about self‐collection] … the misconception has been that you have to use a speculum on yourself.’

ID01

Many felt that cervical screening received less promotion compared to other cancers and women's health issues. As such, there was also limited understanding about the role of HPV in cervical cancer, the purpose of screening and some concerns about the reduced frequency of screening and the later age at which screening commences within Australia's HPV‐based screening programme.

‘…5 years seems like a long time…. I suppose there's evidence, but it used to be what 2 years…. Five seems like quite a stretch in a way’

ID87

For those with a better understanding of screening options, knowledge often came from their professional role, interest in women's health, or personal reproductive health experiences.

### 3.2 Theme 2: Variability in Screening Option Communication and Perceived Absence of Choice

When asked how choices were presented to participants at their most recent screen, fewer than half (19, 44%) recalled being offered a choice between self‐ and clinician‐collected screening.

For those who were offered a choice, the way this was presented varied (Table 3). Some felt that options were presented equally and that the healthcare provider did not try to persuade them about either option. Others felt the healthcare provider either favoured clinician collection or self‐collection. Several participants were only offered self‐collection after they had specifically asked about it.

**Table 3:** Variation in how cervical screening options were presented to screening participants, with supporting quotes.

Theme	Sub‐theme	Example quotes
Offered choice	Options presented equally	‘*she [GP] didn't try and steer me one way or the other … she reassured me that it would be the exact same results’ ID06*
	Clinician‐collected presented as superior	*‘one of the things that she said was sometimes or often with self‐collection if you don't do it right, you have to come back. And the GP has to do it anyway. So I think she sort of angled it a little bit as “if you're happy for me to do it, I might as well just collect it for you…”’ ID02*
	Self‐collection presented as superior	‘*I almost got the impression [that self‐collection] was being encouraged over the GP doing it. And I didn't want that [self‐collection]’ ID87*
	Provided choice after the patient requested	*[Healthcare provider said] ‘of course, I can do that for you right now …she was reflecting really positively on it and had no hesitation to give me what I needed.’ ID46*
Not offered choice	Clinician collection provided with no choice offered	‘*It [self‐collection] was not presented, there was no choice’ ID27*
	Potentially ineligible due to timing, gynaecologist and symptoms	‘*I went to the gynaecologist. not sure maybe 2021 or 2022 … I can't remember’ ID35*
	Denied self‐collection when asked for it	‘*I did ask if I'm eligible for self‐collection, and she made it very clear that self‐collection is not accurate, because people can't get their cervix. And that it's always better just to come to the clinic. So, I just went through with it.’ ID52*
	Discussed previously but patient booked for clinician collection	‘*I could have done it myself, but I was there for other things … he has discussed it with me before’ ID51*
	Self‐collection suggested for the next screen only	‘*after we did that screening, she said, “next time if you want to, we do have the option now for self‐collection, that might be something you'd be interested in”’*

Many participants who were not offered a choice had a clinician‐collected sample taken. However, some of these participants were unsure if they had screened prior to, before or after self‐collection became universally available, or if the consultation was related to follow‐up of a previous abnormal result, meaning that self‐collection may not have been clinically appropriate. This was confirmed for nine participants against the NCSR. Some participants were denied self‐collection when requested, as the healthcare provider believed it to be inferior to clinician collection. Others had discussed self‐collection at previous consultations, or had it presented as an option for their next screen after they were provided with a clinician‐collected test.

Interviewed participants expressed a range of different emotions about not being provided with the choice of self‐collection. This ranged from feeling ambivalent, disappointed, angry or disempowered.

‘I wasn't fazed at the time that I wasn't offered it because I just assumed that it wasn't something that clinic offered. But now that I've discovered that I am eligible, and I probably should have been offered it, now I feel a bit … why didn't I get that choice?’

ID39

### 3.3 Theme 3: The Value in the Choice Itself, in the Unique Attributes of Each Option, and in the Characteristics of the Healthcare Providers Involved in Cervical Screening

Overwhelmingly, participants valued having screening choices, either for themselves or for others. Being provided with a choice was seen to enable people to choose an option that best suited their needs and provide autonomy over their own healthcare.

‘So, I think there is definitely value in there being different options [for cervical screening] that are available. You can kind of pick the one that suits you or suits your needs at the time’

ID21

For many, self‐collection was a favourable option when access was limited to a male, unfamiliar or personally known healthcare provider, particularly in regional and remote areas. Others reported that they would continue to choose clinician collection if they had access to their regular healthcare provider, whom they trust.

‘Having a GP that I trust doing that [clinician‐collection] was fine. If I was in a situation where I didn't have that kind of relationship with someone… I may feel different about a self‐collection option.’

ID02

The features of self‐collection that were valued by participants included increased autonomy and control over their screening experience, convenience and ease, privacy, flexibility of healthcare providers they could see for screening, and perceived opportunities to reduce costs. A commonly reported valued feature of self‐collection was reduced discomfort—both physical and psychological—due to the less invasive nature of the test.

‘I find Pap smears traumatic. And I find this [self‐collection] option so unbelievably better. There is no comparison to me. It is so much less invasive. It is so much less stressful…. There's no pain involved…. To be able to do it and not feel so stressed about it is a real gift.’

ID31

Some indicated that while self‐collection was not their choice, they noted potential benefits for people with histories of trauma, stigma and discrimination in the healthcare system. This was confirmed by a participant from the LGBTQ+ community, who described choosing to specifically travel to an inclusive healthcare provider for self‐collection.

‘When I reflect on what were the barriers or what internally stopped me from doing [cervical screenings] is that it is difficult to access that culturally appropriate service, especially in this sort of region. And once you have that service, you trust that service and rely on them. So both my gynaecologist and GP, I feel very comfortable with them, culturally, I feel very safe. So, I do make the distance [sic]’

ID46

Many participants valued having a choice of where they could perform self‐collection. Benefits of collection in the clinical setting, including clinic bathrooms or privately in consultations room, was the ability to ask their healthcare providers questions and complete the test immediately. For others, benefits of performing self‐collection at home was viewed to provide flexibility, a greater sense of privacy, and potentially removing travel and cost barriers.

‘[An option] where you can [self] collect in the privacy of your own home … makes it [cervical screening] really accessible and takes some of that cost element as well.’

ID02

Many participants also expressed feeling reassured that a second appointment with the healthcare provider could be arranged if they had an abnormal result following self‐collection and that this provided more impetus to engage in screening and follow‐up.

‘It [self‐collection] removes the barriers for people avoiding to get tested [sic]. It's the first step because I think once you do that, you're more likely to go ahead with a speculum exam if you need one’

ID01

Features of clinician collection that participants valued included greater confidence in the accuracy of the result, trust in the skills of the healthcare provider, having a physical examination—particularly for those with previous abnormal results, and the convenience related to the time and cost of not returning for an additional cytology sample if an abnormal result was detected (Figure 1).

‘Just the fact that the first time I did test positive for HPV, so I just wanted to make sure that someone that knew what they were doing, more than me, collected a sample’

ID04

### 3.4 Theme 4—Variation in Participants' Confidence to Self‐Advocate for Preferred Choice and the Influence of Power Dynamics in Clinical Consultations

There were differences in participants' confidence to self‐advocate for their preferred screening method. Several participants who had requested self‐collection but were denied the option felt uninformed about their rights to challenge their healthcare provider. For others, their limited information about self‐collection meant they were more likely to default to the method they were most familiar with, particularly when no choice was offered by their healthcare provider.

‘I had heard about it [self‐collection] quite a while ago…. And I'd forgotten that you could do it yourself at that [appointment] stage. And the GP didn't give me the option … it was just hop on the bed, and away she goes…. Why did I not get the choice?’

ID40

Many felt their confidence to self‐advocate would be influenced by the power imbalances in a medical appointment, including a desire to maintain a good relationship with their healthcare provider or if the provider presented a strong argument for one option.

‘If the doctor thinks it [self‐collection] is a bad idea then maybe it is, but also just not wanting to fight with your health practitioner. So, it's just not worth the argument’

ID21

However, most participants expected to feel more confident advocating for their preferred option in the future once they were better informed. This was reflected by those participants who had a better understanding of their screening options, already feeling more confident during the consultation.

‘I think that's what supported me was having that knowledge already. And when my doctor said to me, are you due for a cervical screening test, I just knew straightaway how I wanted to proceed.’

ID72

### 3.5 Theme 5: Identified Support Needs to Guide Informed Choice and Enact Decisions

Very few participants received information about the accuracy of self‐collection, and the likelihood of being HPV+ and requiring follow‐up, either before or during their medical appointment, with most receiving only a verbal explanation. Many had also not received their reminder letter, and for those who had, many did not recall that it contained information about self‐collection.

‘There might have been [information about self‐collection]. I wouldn't have read it. I just saw the heading that said, you know, that it was due, so I booked it.’

ID87

Most wanted more information to assist with understanding options and felt that cervical screening information should be promoted by healthcare providers as well as in familiar, everyday spaces such as public libraries and sporting clubs.

‘Probably educating doctors, that it [self‐collection] is reliable, and that it's trustworthy, and that the results are real. So that they are happy to promote it within their practices. But maybe out of the box ones, too, like libraries, where you do advertising through that sort of thing’

ID31

Participants called for clear and accessible information to compare their screening options, including step‐by‐step visual guides, links or QR codes for more information and endorsed by credible and trusted sources.

‘A basic rundown of the … pros and cons and ‘how to not‐to’ type things … just a bit of reassurance. it's not too complex … it is a straightforward thing to do’

ID57

Most participants felt that the provision of information about their screening options ahead of the appointment would be beneficial, as it would allow them to feel better prepared and use the appointment to clarify concerns and express preferences.

‘I wish I had seen something before I went to see the doctor … because … I will read it, then I will ask questions, and then make an informed decision.’

ID58

In addition to greater support and resources to make decisions, some wanted structural system‐level changes to access screening options and carry out their choice, such as better access to GPs, different ways to access self‐collection, and reduced medical fees.

‘And I think they should also not be allowed to charge for it [cervical screening tests]. Because that's mainly what put me off is the cost. That more so than the discomfort of doing it [screening].’

ID51

## 4 Discussion

We undertook an in‐depth exploration of the awareness, knowledge and experiences of making cervical screening choices among recently screened individuals in one jurisdiction in Australia. Overall, participants highly valued the availability of the choice between self‐ and clinician‐collected screening. However, most had low levels of knowledge and lacked decision support and access to timely resources to support decision‐making. While many participants place a high level of trust in their healthcare providers and value discussions with them, there were variations in how screening options were presented.

We identified gaps in decisional support needs based on the ODSF and developed recommendations to address these (Figure 3).

Our study revealed that while most people knew of self‐collection, few had accurate knowledge about its eligibility, accuracy and how to access it. Previous studies have shown similar concerns about test accuracy and collecting the sample properly [11, 13], although studies have also shown that screeners feel confident after trying it themselves [26].

Due to knowledge gaps and power imbalances within a clinical consultation, our participants' confidence to self‐advocate for their preferred option was often low. Most participants expected to feel more confident once they were better informed, a theme that has been previously reported [27]. To address these gaps, previous studies have called for greater clarity on where, and how, to access self‐collection, tailored communication on its accuracy, ease and privacy, and reiteration that the option for clinician collection is still available [28, 29].

As identified in our study, people want to see messages about the choices available to them in everyday spaces, in language they can understand, and reiterated within clinical interactions to build their trust and confidence. National campaigns promoting choice and autonomy have been launched since data collection for this study [30, 31]. Post‐campaign evaluations may determine if this need has been addressed.

In addition to empowering screeners to self‐advocate, ensuring healthcare providers are appropriately trained to present options in a balanced way is essential. Despite updated guidelines on self‐collection, a range of healthcare provider‐targeted resources on how to offer choice [32, 33], and accredited online education modules [34], provider adoption of self‐collection into primary care remains varied [17]. Our findings reiterate the need for accessible, clear and targeted communication and education for healthcare professionals that address ongoing concerns about missed opportunities for pelvic examinations, accuracy of self‐collection, and patients' ability to correctly self‐collect, to ensure full‐scale adoption.

Our study found that people's choices were impacted by the value they place on different attributes of each option. The ‘risk’ of a second visit following self‐collection was a disadvantage for some, worried about the cost and the time it would take, while others perceived it as reassuring. Informing people of this possibility could help inform screening decisions based on an individual's values. Decision aids have been shown to improve patient knowledge, reduce decisional conflict, clarify values and increase patient involvement in decision‐making [35].

Our study calls for widespread decision support tools to communicate to all stakeholders the equivalence in accuracy between screening options and key trade‐offs, including explaining how and why they are equivalent (because HPV is shed from the cervix into the vagina if an infection is present). A simple one‐page infographic could be used to highlight these key areas for all people considering their cervical screening options, with provision of links (via QR codes or to a website) or additional hard copy resources for those who want to know more. Recently, a cervical screening decision aid was developed and tested with 360 women attending a Gynaecology Outpatient Clinic within the Australian context [36], but it did not include information on the risk of a return visit for those who test positive on a self‐collected swab. A Canadian online preference‐elicitation tool for cervical screening modalities, based on decision‐making concepts and validated against the ODSF, found preliminary evidence supporting participants in identifying informed, values‐based cervical screening preferences [27]. Additionally, a recent UK study developed a comprehensive decision support strategy in preparation for the introduction of the choice of self‐collection within their screening programme [37]. These existing resources could be adapted to the Australian context to support potential screeners in understanding their options and being active in their decision‐making.

We found inconsistencies in participants receiving reminder letters and missed opportunities to raise awareness of self‐collection. Reminder systems to alert people they are due for screening provide an opportunity to improve knowledge of options before consultations. Australia's NCSR has recently launched a participant portal, which could be more widely promoted [38]. This could further facilitate participants' autonomy to check their own screening history and become informed about screening options.

Participants felt self‐collection would be more advantageous if it could reduce appointment costs and be more accessible. Australia's guidelines support flexibility in where self‐collection can be offered, and by which healthcare provider [39], enabling nurses and Aboriginal health workers/practitioners who are highly trusted by the community [40, 41] to discuss screening options. New funding models to allow these professionals to independently request and be reimbursed by Medicare, alongside access to the NCSR, could support information provision and outreach initiatives, a key enabler to engaging under‐screened populations [42]. The development of integrated pathways to ensure continuity of care, where referral is needed for those testing positive for HPV, is now a policy priority.

To address these decisional gaps and support needs, we recommend four key actions:

1. Build on existing national campaigns by making tailored resources available through channels relevant to different communities to address inadequate knowledge and support informed decision‐making.
2. Develop decision support tools that clearly articulate key trade‐offs between screening options.
3. Monitor the roll out of updated training resources for healthcare providers, to ensure consistent presentation of screening options in line with NCSP guidelines.
4. Improve system accessibility by reducing appointment costs, supporting flexible ways to access self‐collection and exploring new funding models to promote broader workforce involvement in screening, enabling people to enact the decisions they make.

### 4.1 Strengths and Limitations

Interviews informed by the ODSF helped to explore key decisional needs influencing the experiences and choices of cervical screening. Our study found reasonable accuracy of the self‐reported data via NCSR validation, despite the limited number of variables permitted to be used for matching screening histories. Limiting participants to those 1 year post previous screen was intentional to reduce recall bias. The study was not promoted as a study about ‘self‐collection’ but of cervical screening, which reduced the risk of bias in recruiting those offered or not offered the choice.

Our study sample were highly educated people who mostly spoke English as a first language, only a small number identified as LGBTQI+ and just over a quarter lived outside a metropolitan area which limits the generalisability of our findings.

## 5 Conclusion

Australia's national guidelines for cervical screening emphasise providing people with an informed choice of screening options. Overwhelmingly, participants valued the inclusion of the choice between self‐ and clinician‐collected screening, as this was seen to empower decision‐making and allow people to choose the option that suited them best.

However, we found that very few people were making informed choices that aligned with their values because they had low levels of knowledge, felt uninformed about options, did not have an accurate perception of their risk or outcomes of each screening modality (such as efficacy and likelihood of being HPV+) and many were not participating in decision‐making because this was being controlled by the healthcare provider (either by not providing them with options, attempting to sway them towards a particular option, or denying them an option when requested). A range of strategies are needed to ensure screeners feel empowered, supported and informed to make and carry out a real choice, including widespread promotion, decision support tools, appropriately training healthcare providers and system improvements.

## Author Contributions

**Ana Machado Colling:** conceptualisation, writing ‐ original draft, methodology, writing – review and editing, formal analysis, project administration, data curation, software, visualisation. **Tessa Saunders:** conceptualisation, methodology, writing – review and editing, formal analysis, data curation, software, visualisation, investigation. **Nicola Creagh:** writing – review and editing, data curation, software. **Maleeha Ashfaq:** formal analysis, writing – review and editing, software, investigation. **Julia Brotherton:** conceptualisation, investigation, writing – review and editing, supervision, methodology. **Claire Nightingale:** funding acquisition, writing – review and editing, supervision, conceptualisation, investigation, methodology.

## Ethics Statement

Ethical approval was received by the University of Melbourne Human Research Ethics Committee (HREC number: 2025‐27551‐65265‐7).

## Consent

Participants gave informed consent before taking part in the study.

## Conflicts of Interest

J.B. was previously employed at the Australian Centre for the Prevention of Cervical Cancer. ACPCC has received donations of equipment and HPV test kits from Roche, Seegene, Abbott, BD, Cepheid and Copan for research and validation studies. She is also an investigator on the SHE‐CAN HPV‐based cervical screening trial in India, which has accepted donations of consumables and tests from Copan, Abbott and Seegene.

## Data Availability

The datasets generated during the current study are available from the corresponding author upon reasonable request and HREC approval conditions.

## Endnotes

1 According to 2023 estimates, the current risk of requiring a return visit for non‐16/18 HPV is approximately 5.6% [43].
